# Genetic characterization and passage instability of a novel hybrid virulence plasmid in a ST23 hypervirulent *Klebsiella pneumoniae*

**DOI:** 10.3389/fcimb.2022.870779

**Published:** 2022-07-28

**Authors:** Lin-Ping Fan, Yang Yu, Shanshan Huang, Wenjian Liao, Qi-Sen Huang, Fang-Ling Du, Tian-xin Xiang, Dan Dan Wei, La-Gen Wan, Wei Zhang, Yang Liu

**Affiliations:** ^1^ Department of Clinical Microbiology, First Affiliated Hospital of Nanchang University, Nanchang University, Nanchang, China; ^2^ School of Public Health, Nanchang University, Nanchang, China; ^3^ Department of Respiratory and Critical Care Medicine, First Affiliated Hospital of Nanchang University, Nanchang University, Nanchang, China; ^4^ Department of Infectious Disease, First Affiliated Hospital of Nanchang University, Nanchang University, Nanchang, China

**Keywords:** hypervirulent *Klebsiella pneumoniae*, type IV secretion systems, conjugative virulence plasmid, toxin-antitoxin systems, whole-genome sequencing, fitness cost

## Abstract

Hypervirulent variants of *Klebsiella pnuemoniae* (hvKP), which causes life-threatening infections, is a global priority pathogen and frequently harbours virulence plasmids. The virulence plasmids have emerged as the predominant vehicles carrying the major pathogenic determinants of hypermucoviscosity and hypervirulence phenotypes. In the present study, we characterized a novel virulence plasmid in AP8555, an ST23 hvKP strain, which induced a metastatic infection and fatal septic shock in a critically ill patient. The serum killing assay, the quantitative biofilm formation assay, the *G.mellonella* infection model, and the mouse lethality assay demonstrated that AP8555 was almost as virulent as the hvKP strain NUTH-K2044. The plasmid pAP855 could be conjugated to *Klebsiella quasipneumoniae* ATCC700603 and *E. coli* J53 at a frequency of 7.2× 10^−5^ and 8.7× 10^−7^, respectively. Whole-genome sequencing and bioinformatics analysis confirmed that the plasmid was novel, clustered to the incompatibility type of IncHI1B/IncFIB/IncFII and presented high similarity to the pK2044 plasmid. In contrast, a 130-kb large-fragment insertion was observed on the plasmid, which introduced a genetic hybrid zone with multiple conjugation-related genes of type IV secretion systems (T4SS) and *CcdAB* toxin-antitoxin systems (TAS) to the plasmid. In the transconjugants, the presence of pAP855 had a negative impact on bacterial fitness, but enhancing the virulence-associated phenotypes. *In vitro* evolution experiments showed that pAP855 in the transconjugants could not be stably inherited after 10 days of passage. Our study not only reports a novel hybrid plasmid but also highlights the putative pathway of conjugative virulence plasmid formation and evolution by means of genetic rearrangement through sequence insertion. These findings indicate that structural versatility could contribute to the dissemination of cointegrate virulence plasmid, although the plasmid incurred a fitness cost. Therefore, continuous monitoring the acquisition of conjugative virulence plasmids may have critical value for plasmid research and increase awareness of hvKP.

## Introduction

Hypervirulent *Klebsiella pneumoniae* (hvKP), which causes severe infections such as pyogenic liver abscesses, meningitis, endophthalmitis, and necrotizing fasciitis, has been increasingly reported in China (Zhu et al., 2021). The majority of the reported hvKP strains belong to capsular types K1/K2 and harbour virulence plasmids, which account for their strong anti-phagocytic abilities and serum resistance (Yeh et al., 2012; Shi et al., 2018; Du et al., 2019). The virulence plasmid in the NTUH-K2044 strain that has been named pK2044 was recently found to carry major virulence genes, notably capsular polysaccharides regulator genes (*rmpA* and *rmpA2*) and those encoding siderophores (e.g., *iroBCDN*, *iucABCD*, and *iutA*) and was not a conjugative plasmid (Wu et al., 2009). These virulence genes have been recognized as essential contributors to the virulence of hvKP and may serve as potential biomarkers for hvKP (Hsieh et al., 2008; Hsu et al., 2011). The loss of virulence plasmid is correspondingly associated with a significant decrease in virulence (Nassif and Sansonetti, 1986; Nassif et al., 1989). Thus, research has confirmed that a correlation exists between the carriage of virulence plasmids and the hypervirulent phenotype (Tang et al., 2010).

Virulence plasmids are important drivers of hvKP evolution and facilitate the acquisition of virulence genes in a single-transfer event. In recent years, the emergence of fusion/hybrid plasmids has been frequently reported in hvKP isolates, causing serious public concern (Yang et al., 2019; Zhang et al., 2020). Unlike traditional plasmids, fusion/hybrid virulence plasmids possess more insertion sequences with high plasticity, playing a crucial role in the dissemination of virulence genes (Wyres et al., 2019). In this study, we reported a novel conjugative virulence plasmid pAP855 and assessed the fitness cost and stability in order to systematically evaluate the transmission potential of this plasmid.

## Materials and methods

### Patient and isolates

In April 2019, a 58-year-old male patient was admitted to the first affiliated Hospital of Nanchang University with an intra-abdominal abscess. The patient had type 2 diabetes mellitus, diagnosed 7 years earlier, and had not travelled outside the city within the 3-month period preceding this admission. The patient had a high fever (41°C) and breathing difficulty and was transferred to the intensive care unit (ICU) after developing septic shock induced by the abdominal infection. An anti-infection treatment of tigecycline, meropenem, and ciprofloxacin was administrated to this patient. However, the treatment was unsuccessful, and the patient eventually died of sepsis caused by *K. pneumoniae*.

AP8555, a *K. pneumoniae* strain, was isolated from the patient’s blood sample. It was identified as a strain of *K. pneumoniae* using the ANI (http://enve-omics.ce.gatech.edu/ani/). The result of string test demonstrated the isolate was characterized as hypermucoviscous (**Supplementary Figure S1**).

### Antimicrobial susceptibility testing

Minimum inhibitory concentrations of the commonly used antimicrobials were determined by the Vitek 2 system in accordance with the Clinical and Laboratory Standards Institute guidelines (CLSI, 2020). Additionally, the activity of tigecycline was detected *in vitro* using the E-test method, which is based on the standard prescribed by the US Food and Drug Administration. *Escherichia coli* ATCC25922 and *K. quasipneumoniae* ATCC700603 were used as quality control reference strains for antimicrobial susceptibility testing. ESBL-producing *K. quasipneumoniae* ATCC700603 was resistant to cephalosporins commonly used in clinical practice but susceptible to carbapenems and tigecycline. The ESBL encoding genes *blaOXA-2* and *blaSHV-18* was in the plasmid pKQPS2 (Protocols of mouse experiments were approved by the ethics committee of the first affiliated Hospital of Nanchang University, Jiangxi, China). The detailed minimum inhibitory concentrations for *K. quasipneumoniae* ATCC700603 are shown in Table S1.

### Virulence assessment of hypervirulent *K. pneumoniae*

#### (1) Serum killing assay

Serum bactericidal activity was assessed using a method that has been previously described (Wei et al., 2016). Briefly, human blood was extracted from 10 healthy individuals and the serum was then stored at -80°C. An inoculum containing 10^6^ CFU of bacteria was mixed with 75 μl of pooled normal human serum in microtiter plates and then incubated at 37°C for 3h. Resistance grading was scored using six grades as previously described (Liu et al., 2019), with Grades 5 and 6 indicating high-grade serum resistance. The experiment was repeated three times.

#### (2) Mouse lethality assay

The virulence of hvKP AP8555 was assessed by infecting mice intraperitoneally, as previously described (Liu et al., 2019). Five-week-old female BALB/c mice obtained from the Nanchang University Animal Centre were used for the virulence assessment. A ten-fold serial dilution was performed to obtain the appropriate concentration of 10^6^-10^3^ Colony-Forming Units (CFU)/mL for determining the median lethal dosage (LD50). One hundred microliters of each concentration were injected into the BALB/c mice intraperitoneally. Daily records were maintained of all of the surviving inoculated mice. The *K. quasipneumoniae* ATCC700603 and the hvKP NTUH-K2044 were respectively used as low and high virulence controls.

#### (3) *Galleria mellonella* infection model

The virulence was assessed in *Galleria mellonella* larvae, each weighing approximately 300 mg (purchased from Tianjin Huiyude Biotech Company, Tianjin, China) by injecting 10 larvae with concentrations of 1 × 10^6^ CFU/ml bacteria per 10 microliter aliquot using the methods described in a previous study (Gu et al., 2018). The survival rates of *G. mellonella* were recorded over a 48h period post-infection. Control animals were injected with phosphate-buffered saline (PBS). The hvKP NTUH-K2044 and *K. quasipneumoniae* ATCC700603 were used as controls of high and low virulence strain, respectively. Statistical analyses were performed and visualized with GraphPad Prism v. 7.00 (GraphPad Software Inc., La Jolla, CA, USA).

#### (4) Quantitative biofilm formation assay

A 96-well polystyrene microtiter plate was used for conducting a quantitative evaluation of biofilm formation measured by the crystal violet staining of cells cultured in Luria-Bertani (LB) broth, as previously described (Pal et al., 2019). The test was performed in triplicate. Briefly, the clinical isolates and control strains were inoculated in 10 mL of LB and incubated at 37°C for 18h. The individual wells in the 96-well polystyrene microtiter plate were filled with 150 μL of bacterial culture (1.5 × 10^7 ^CFU/mL). The microtiter plate was incubated at 37°C for 18h. The wells were then washed three times to remove free-floating planktonic bacteria and measured by conducting 0.5% crystal violet solution staining for 20 min. As a final step, 95% ethanol was added to each well to elute the biofilm-bound dye. The data on biofilm formation were quantified by performing optical density at 540nm (OD_540_) measurement. The average was then calculated for each bacterial strain. The NTUH-K2044 strain, which exhibited strong biofilm formation, served as the positive control.

#### (5) Growth curve measurements

Overnight cultures of plasmid-free and plasmid-carrying strains were diluted to an optical density at 600 nm (OD_600_) of 0.05, and the diluents were grown at 37°C for 12h with vigorous aeration (200 rpm) in an antibiotic-free LB broth. The culture cell density was determined every hour and measured at OD600. All experiments were repeated 3 times. The growth curves were analysed by GraphPad Prism version 6 using one-way analysis of variance (ANOVA).

### DNA sequencing and data analysis

The genomic DNA of the AP8555 strain was extracted using the QIAamp DNA Mini Kit (Qiagen, Germany) and subsequently underwent sequencing and assembly performed by Shanghai Yuanxu Biotechnology Co. Ltd., as described in a previous study (Liu et al., 2019). A total of 5 µg genomic DNA was sheared by g-TUBE (Covaris, US). The sequencing library with 10-kb size was constructed using the standard PacBio RS sample preparation instructions and then sequenced on Pacific Biosciences RS II sequencing platforms (Pacific Biosciences, US). Additionally, a 300-bp paired-end library from the same genomic DNA was prepared according to Illumina TruSeq DNA sample preparation recommendations and sequenced on Hiseq 2500 platforms (Illumina, US) with a read length of 150 bp. The PacBio data (10 kb fragment library, 356,001 reads) were assembled using Hierarchical Genome Assembly Process (HGAP) software (Chin et al., 2013), generating a one-contig genome and a plasmid. The assembled genome and plasmid from the PacBio data were further proofread using Hiseq data *via* Bowtie2 and samtools (Li et al., 2009; Langmead and Salzberg, 2012). Finally, a whole genome assembly without redundancy was obtained.

### Bioinformatic analysis of sequenced plasmids of *K. pneumoniae*

Gene prediction for strain AP8555 was conducted using glimmer 3.02 (Delcher et al., 2007). Sequence comparison was performed the BLAST analysis (http://blast.ncbi.nlm.nih.gov/Blast.cgi) and the BLAST Ring Image Generator v.0.95.22. Pathways involved in the genes were constructed by the use of Kyoto Encyclopedia of Genes and Genomes (KEGG) (Kanehisa and Goto, 2000). Gene COG was classified according to the conserved domain database (Marchler-Bauer et al., 2015). The STs and Capsular typing were determined by online tool (https://bactopia.github.io/bactopia-tools/kleborate/). The acquired antimicrobial resistance genes were identified by uploading assembled genomes to the Resfinder server v2.11. Putative virulence factors were predicted by VRprofile with the BLASTp-based Ha-value > 0.64, which collected 2,454 virulence factors from the Virulence Factors Database (VFDB). The genome was analyzed for the presence of prophages using PHAST (Zhou et al., 2011). The plasmid replicons databases were obtained from the Center for Genomic Epidemiology (http://www. genomicepidemiology.org/). According to the *in silico* analysis, the 28-bp fusion site was identified using the matchPattern function in the Biostrings R package with the specific sequence ‘AGATCCGNAANNNNNNNNTTNCGGATCT ‘.

### Nucleotide sequence accession numbers

The genome sequence of *K. pneumoniae* AP8555 was available under the accession numbers CP035383.1 (AP8555 chromosome) and CP035384.1 (plasmid pAP855). The genome sequence of NTUH-K2044 was available under the accession numbers CP026011.1 (NTUH-K2044 chromosome) and CP026012.1 (plasmid pK2044). The genome sequence of *K. quasipneumoniae* ATCC700603 was available under the accession numbers CP014696 (ATCC700603 chromosome), CP014697 (plasmid pKQPS1), and CP014698 (plasmid pKQPS2) (Elliott et al., 2016).

### Conjugation experiments

A plasmid conjugation assay was performed using *K. pneumoniae* AP8555 as the donor strain and *K. quasipneumoniae* ATCC700603 (cefazolin resistant), *E. coli* J53 (sodium azide resistant) as the recipient strain. The experiment was modified as described in a previous study (Wei et al., 2016). About 1 × 10^8^ CFU of both donor strain and recipient strains were mixed and dotted on sterilized filter paper, which was then incubated on an LB agar plate for 18 h at 37°C. Transconjugants were selected by LB agar plates containing 32 mg/liter cefazolin-5 mg/liter tellurite or 100 mg/liter sodium azide-5 mg/liter tellurite. A polymerase chain reaction (PCR) test for detecting *rmpA/rmpA2* was performed on the transconjugants. The conjugation efficiency was calculated by dividing the number of transconjugants by the number of recipient cells (Xu et al., 2021).

### Southern blot analysis

S1-Pulsed Field Gel Electrophoresis (S1-PFGE) was performed to analyze the number and size of plasmids in the hvKP AP8555 strain and transconjugants based on the criteria proposed by Tenover et al. (Tenover et al., 1980). Furthermore, the AP8555 isolate and transconjugants underwent southern blot hybridization to determine whether they contained virulence plasmid. Plasmid DNA from the isolates was digested with S1 nuclease and separated with the PFGE. The plasmid DNA fragments were then transferred to a positively charged nylon membranes. Southern blot hybridization was performed using a DIG-labelled *rmpA2*-specific probe to confirm the presence of the virulence plasmid in the AP8555 strain (Gu et al., 2018).

### Pairwise competition assay

To assess the cost of carriage of the cointegrate plasmid, pairwise competition experiments between plasmid-bearing strains and their plasmid-free isogenic ancestors were performed in antibiotic-free LB broth. Briefly, colonies from each strain were diluted to 0.5 McFarland standard and mixed at a ratio of 1:1 in 5 mL LB broth at 37°C for 72h with shaking. Every 24h, 5μL cultures were reinoculated in 5 mL of fresh LB medium. The number of cells for each strain was determined by spreading serial 10-fold dilution onto LB agar plates and LB agar plates with 5 mg/L of potassium tellurite. Potassium tellurite can enhance the fitness of bacteria, and has resistance to oxidative, genotoxic, heavy metal, proton motive force, cell wall, membrane, phage, and protein synthesis stress, which ensure the stability of pathogens. pAP855 is resistant to potassium tellurite, so AP8555 can grow steadily, while ATCC700603 and *E.coli* J53 are not. All competition experiments were performed a minimum of three times. The relative fitness was calculated as follows: *w* = ln(NRt/NR0)/ln(NSt/NS0), where NR is the number of resistant clones and NS is the number of susceptible clones, with values below 1 indicating the fitness cost.

### Plasmid stability experiments

To evaluate the stability of the cointegrate plasmid in the transconjugants, the cointegrate plasmid-bearing ATCC700603 and J53 were propagated by serial passaging for 10 days in antibiotic-free LB broth under shaking (200 rpm) at 37°C. Two microlitres of overnight culture was collected and used for inoculation at a 1:1,000 dilution every 12h. Plasmid stability was calculated every 5 days by counting the number of colonies that grew on LB agar plates and potassium tellurite-containing plates. Biological triplicates and technical triplicates were completed in this experiment.

### Ethics statement

The study has been evaluated by the Ethics Committee of the First Affiliated Hospital of Nanchang University. Patients involved in the study were anonymized, no informed consent was acquired because of the retrospective study.

## Results

### Isolation of a hypervirulent *K. pneumoniae*

A hypervirulent *K. pneumoniae* isolate (termed AP8555) at >10^6^ CFU/mL was recovered from the patient’s blood sample. The AP8555 were sensitive to all of the antibiotics recommended by the Clinical and Laboratory Standards Institute for the susceptibility testing of *Enterobacteriaceae* apart from its intrinsic resistance to ampicillin. The detailed minimum inhibitory concentrations for AP8555 are shown in **Supplementary Table S1**.

### Virulence assessment of hypervirulent *K. pneumoniae* AP8555

**Figure 1:**
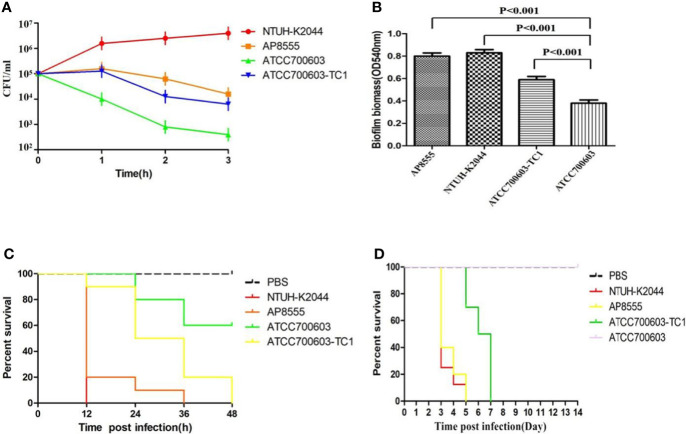
Virulence assessment of the strain AP8555 and the reference strains. **(A)** Serum resistance level of the *Klebsiella pneumoniae* AP8555 strain and the reference strains. The *K. pneumoniae* AP8555 strain and the hypervirulent *K. pneumoniae* (hvKP) NUTH-K2044 strain exhibited grades 4 and 6 responses, respectively, whereas *K. quasipneumoniae* ATCC700603 exhibited a grade 1 response. **(B)** Biofilm biomass was expressed as crystal violet optical density (OD_540nm_). Data were expressed as means ± SD (error bars) of results for at least three independent experiments for each strain. **(C)** The effect of 1 × 10^6^ CFU of each *K. pneumoniae* strain on the survival of *G. mellonella*. NUTH-K2044 was used as a positive control, and ATCC700603 was used as a negative control in these studies. **(D)** Kaplan–Meier survival curves for *K. pneumoniae* AP8555-infected mice. Mice were intraperitoneally infected with 10^3^ CFU of different *K. pneumoniae* strains. The hvKP NUTH-K2044 strain, *K. quasipneumoniae* ATCC700603 strain, and saline were used in controls. The virulence of AP8555 did not differ significantly from that of NUTH-K2044 (P > 0.5, by log-rank test). No mice in the ATCC700603 or saline groups died during the 14 days of observation.

The serum killing assay (**Figure 1A**), the quantitative biofilm formation assay (**Figure 1B**), the *G. mellonella* infection model (**Figure 1C**), and the mouse lethality assay (**Figure 1D**) revealed that hvKP AP8555 strain was almost as virulent as the hvKP NUTH-K2044 strain. Both strains were hypervirulent compared with the ATCC 700603 strain.

### Genome sequencing and analysis

The AP8555 strain possessed a chromosome and a virulence plasmid that were respectively 5.46 Mb and 357,837 bp in size (**Supplementary Table S2**). Multilocus sequence typing revealed that the AP8555 strain was ST23 (gapA-infB-mdh-pgi-phoE-rpoB-tonB, allele no. 2-1-1-1-9-4-12), and the K1 serotype was confirmed through capsular typing *via* the *wzi* allele (https://bactopia.github.io/bactopia-tools/kleborate/). **Supplementary Figure S2A** presents a summary of the genomic features of AP8555. BLAST visualization of the NTUH-K2044 genome showed highly similar to genome content of AP8555, with only a few regions remaining unidentified in AP8555 (**Supplementary Figure S2B**). Antibiotic resistance genes (*fosA*, *bla_SHV-19_*, *oqxA*, *oqxB*) were found in the chromosome of AP8555, while no antibiotic resistance genes were found in the plasmid of the AP8555 strain. A total of 182 putative virulence genes belonging to nine virulence gene clusters were detected on the AP8555 chromosome, which contains almost all the identified virulence genes of hvKP strains (Wang et al., 2018). A range of putative virulence genes was also located in the virulence plasmid pAP855 with IncHI1B/IncFIB/IncFII replicons, including *iroBCDN, iucABCD, rmpA, rmpA2*, and *iutA*.

### Overall sequence analysis of pAP855

**Figure 2:**
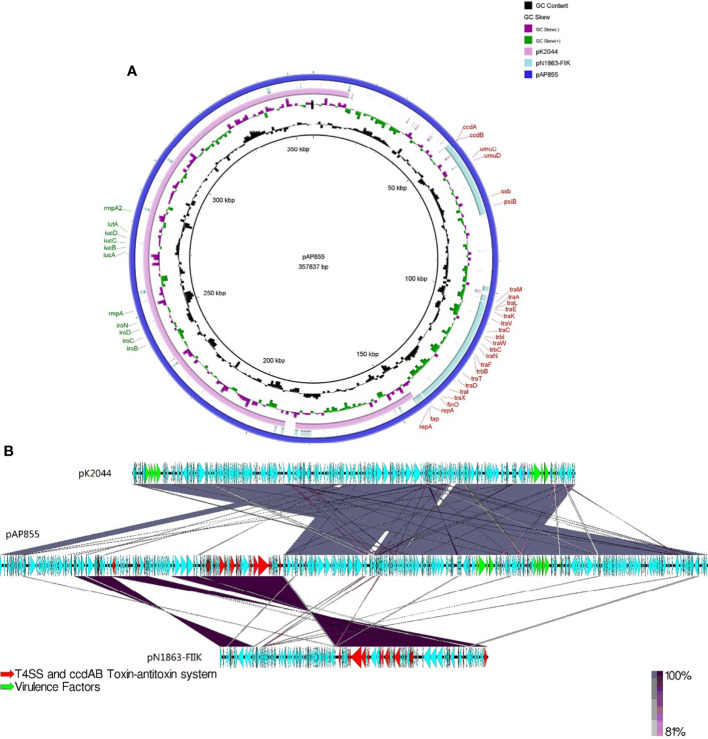
Sequence lignment of conjugative virulence plasmids: pAP855 and pK2044 pK2044 virulent plasmid, IncHI1B/IncFIB; pN1863-FIIK plasmid, IncFII; pAP855 virulent plasmid, IncHI1B/IncFIB/IncFII. **(A, B)** are plasmid comparison diagrams, **(A)** is a circle diagram, and **(B)** is a line diagram.

The complete nucleotide sequence of pAP855 has been determined. The plasmid has an average G+C content of 42.3%. The bioinformatic analysis revealed 232 ORFs with potential coding capacity and an ATG start codon. Compared with the virulence plasmid pK2044, besides the classical IncHI1B/IncFIB virulence plasmid fragment, pAP855 included a 130-kb fragment from a conjugative IncFII(K) plasmid, which exhibited 70% coverage and 99.79% identity with a conjugative plasmid pN1863-HI2 (Accession No. NZ_MF344583.1) carrying the *bla*_KPC-2_ gene from a *Enterobacter cloacae* strain N1863 from China isolated in 2017. As shown in **Figure 2**, this region contained a *CcdAB* toxin-antitoxin system (TAS) and multiple conjugation-related genes of F-type type IV secretion systems (T4SS). Furthermore, the 130-kb region contain T4SS gene clusters and the *oriT* region, have two specific 28-bp fusion site, which indicates that the virulence plasmid pAP855 can be considered mobilizable.

### The *tra* region encoding transfer proteins are essential for conjugation

ORFs spanning bp 102210 to 142348 in pAP855 shows a *tra* region which probably encode a conjugative transfer system. The *tra* region of pAP855 is organized like and highly homologous to the *tra* regions of F-like plasmids belonging to the type IV family of secretion systems. Analysis of the pAP855 *tra* region revealed 26 putative ORFs. Genes coding for the conjugal transfer system spanned approximately a quarter (42 kb) of the plasmid. The gene products can be classified into the following four blocks based on functions inferred from their closely related homologs: pilus biogenesis (*traM, traA, traL, traE, traK, traB, traV, traC, traW, traU, traF, traH, traS, traX, traQ, traG*, and *traT)*; and the regulatory fertility inhibition protein (*FinO*); DNA nicking and initiation of transfer (*TraI* and *TraD*); and mating aggregate stabilization (*TraN* and *TraG*); and mating pair formation (*trbB, trbF, trbI, trbC, and trbE*).

### Plasmid maintenance is accomplished by a toxin-antitoxin addiction system

Next to the putative *rep* gene of pAP855, there are two ORFs which apparently belong to a plasmid type II toxin-antitoxin system *CcdAB*. The *ccdA* gene encodes antitoxin. The *ccdB* gene encodes for a gyrase inhibitor toxin that kills the cell in the absence of the *CcdA* antitoxin, demonstrating this system is active. The well-characterized plasmid based *CcdAB* TA system is important for plasmid maintenance (Wu et al., 2020). Stable maintenance of pAP855 might also be achieved by the toxin-antitoxin system responsible for postsegregational elimination of plasmid-less cells from a bacterial population.

Moreover, the virulence plasmid pAP855 contains a umuDC operon that have been extensively characterized for their role in SOS mutagenesis. The virulence plasmid pAP855 also encodes the antirestriction proteins ArdA (alleviation of restriction of DNA) that specifically affect the restriction and modification activities of type I systems, which may give some advantage for efficient transmission of the virulence plasmid pAP855. Thus, further work is required to confirm their roles in plasmid stability.

### Conjugation experiments

**Figure 3:**
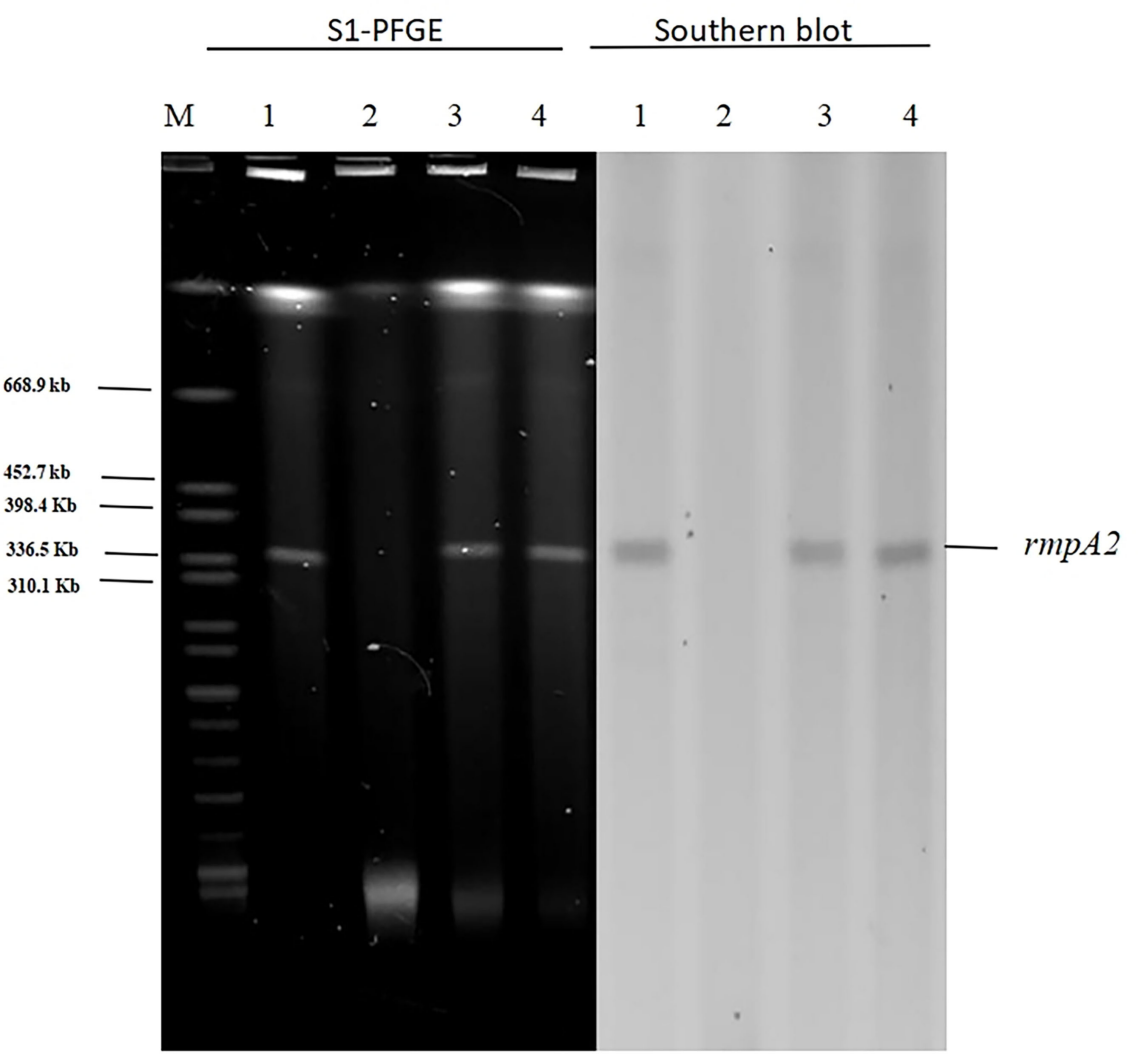
S1-PFGE and Southern hybridization of the virulence plasmid (marker gene rmpA2) 1: AP8555 strain; 2: K. quasipneumoniae ATCC700603; 3: K. quasipneumoniae ATCC700603 -TC1; 4: E. coli J53-TC1.

In light of our finding that pAP855 carries multiple conjugation-related genes, we performed conjugation experiments to test its potential to be transferred to *K. quasipneumoniae* ATCC700603 and *E. coli* J53. The results for S1-PFGE and southern blot hybridization revealed that the plasmid pAP855 from the hvKP AP8555 strain was successfully transferred to the recipients, *K.quasipneumoniae* ATCC700603 and *E.coli* J53 (**Figure 3**) by conjugation experiments. We then knocked out the *traE* gene that encodes an essential component of T4SS responsible for seeding the site of pilus assembly. *traE*-deficient pAP855 failed to mobilize to *E. coli* J53. The data showed that the virulence plasmid pAP855 could be mobilized, supporting the bioinformatic prediction that its conjugation system is functional (data not shown). Notably, the plasmid pAP855 could be conjugated to *K. quasipneumoniae* ATCC700603 and *E. coli* J53 at a frequency of 7.2×10^−5^ and 8.7×10^−7^, respectively. Conversely, no pK2044 plasmids were successfully transferred to the same recipient.

To determine whether pAP855 acquisition could significantly increase the virulence, we tested transconjugants (*K. quasipneumoniae* ATCC700603-TC1) for the expression of a hypermucoviscosity phenotype. Our data showed that acquisition of the plasmid pAP855 could lead to a significantly increased level of mucoviscosity in transconjugant compared with the level in *K. quasipneumoniae* ATCC700603 (**Supplementary Figure S3**). The virulence level of the transconjugants *K. quasipneumoniae* ATCC700603-TC1 increased dramatically, resulting in a 0% survival rate among the tested *G. mellonella* larvae at 48h, which was significantly lower than the 60% survival rate recorded for those with the recipient strain *K. quasipneumoniae* ATCC700603 (**Figure 1C**). Furthermore, when mice were infected with 5.0 × 10^5^ CFU *K. quasipneumoniae* ATCC700603-TC1, the mortality rate was 100% on day 7. By contrast, no mice in the ATCC700603 or saline groups died during the 14-day test period (**Figure 1D**). Biofilm formation assay revealed that ATCC700603 had significantly increased biofilm formation ability after plasmid acquisition (*P* < 0.5) (**Figure 1B**).

### The biological feature and stability of plasmid pAP855

**Figure 4:**
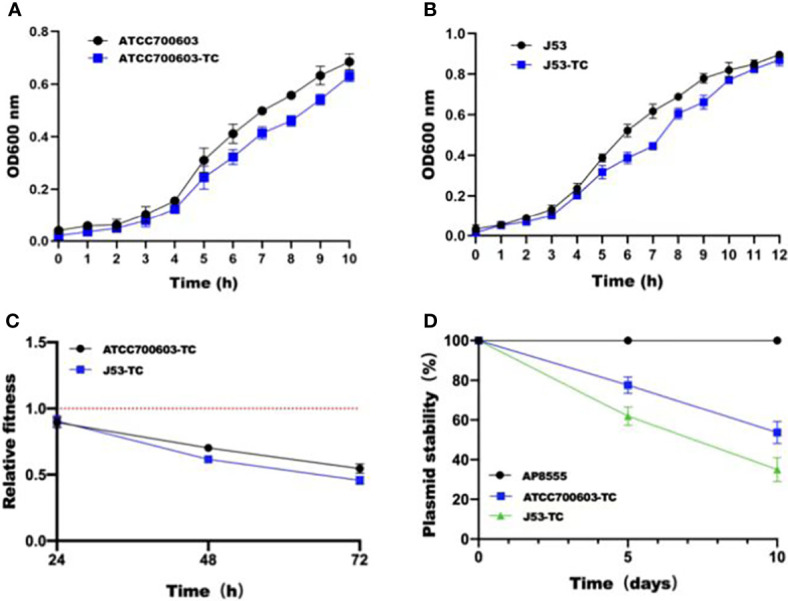
Initial fitness cost after acquisition of cointegrate plasmid in ATCC700603 and J53 strains and plasmid persistence results of pAP855. **(A, B)** Growth curves of *K. quasipneumoniae* ATCC700603 and *K. quasipneumoniae* ATCC700603-TC1, *E. coli* J53 and *E. coli* J53-TC1. **(C)** Relative fitness of two pAP855 carrying strains. Relative fitness value less than 1 indicates fitness defect, and a value greater than 1 indicates fitness benefit. **(D)** Plasmid stability of pAP855 in bacterial population after serial passaging in antibiotic-free LB broth. The experiment was repeated in triplicate. Representative results of three independent experiments are shown, and the data are the mean standard deviation (SD). *P*<0.0001 (one-way ANOVA).

In order to estimate the stability of pAP855 in its natural host AP855, as well as in *K.quasipneumoniae* ATCC700603 and *E.coli* J53, we performed experimental passage experiments under antibiotic-free conditions. pAP855 could be stably maintained in its natural host in at least 95% of cells. However, the plasmid pAP855 was extremely unstable in *K.quasipneumoniae* ATCC700603 and *E.coli* J53, since rapid decline of the plasmid was observed within 5 days (**Figure 4C**). Both partitioning systems and TA systems were observed in the reorganized plasmid pAP855.

According to the bacterial growth curves, we observed that the plasmid caused an obvious decrease in growth rate of *K.quasipneumoniae* ATCC700603 and *E.coli* J53 (**Figures 4A, B**). Pairwise competitions were conducted between plasmid-bearing strains and their plasmid-free isogenic ancestors. The relative fitness of both *K.quasipneumoniae* ATCC700603 and *E.coli* J53 was less than 1, suggesting that the plasmid imposed a fitness cost on *K.quasipneumoniae* ATCC700603 and *E.coli* J53 (**Figure 4D**).

## Discussion

Recently *K. pneumoniae* ST23 (serotype K1) strain that exhibits the hypervirulent phenotype has been recognized as an important pathogen responsible for severe hospital-acquired invasive infections in critically ill patients (Lin et al., 2018). Present studies have shown that pLVPK-like virulence plasmids play a major role in hvKP pathogenesis (Shen et al., 2019). Moreover, it has been shown that the acquisition of the virulence plasmid in *K. pneumoniae* strains results in hypervirulence compared with that of ST11 classic *K. pneumoniae* strains (Dong et al., 2018). The majority of these plasmids have been identified as IncHI1B/IncFIB type plasmids that harbour *rmpA/rmpA2*, *aerobactin*, and *salmochelin*, thereby greatly enhancing the virulence. An ST23 strain lacking pLVPK was found to have significantly reduced virulence, indicating that this plasmid plays an important role in the hypervirulence of *K. pneumoniae* (Lin et al., 2011). Furthermore, virulence plasmids were associated with increased hvKP virulence in animal models (Nassif and Sansonetti, 1986). Moreover, *in vitro* and *in vivo* studies have characterized large plasmids associated with virulence in hvKP strains, which are required for establishing distinct disease phenotypes (Tang et al., 2010).

Typically, pLVPK-like plasmids are not self-transmissible due to the lack of a complete conjugation module. However, plasmid fusion is increasingly detected in hvKP isolates and virulence factors on pLVPK-like plasmids could be integrated into a conjugative plasmid (Dong et al., 2018). Mobile elements are the main contributors to plasmid replicon fusion. In recent years, the emergence of fusion/hybrid virulence plasmids has been frequently reported. The first conjugative virulence plasmid from a *K.variicola* strain, which formed as a result of the integration of a 100-kb fragment of pLVPK-like plasmid into a conjugative IncFIB plasmid, could be transferred to different types of *Klebsiella* strains and increases the virulence (Yang et al., 2019). Another recent study reported a IncHI1 pLVPK-like virulence plasmid in ST11 clinical *Klebsiella pneumoniae* strain can be conjugative to *E. coli* strain EC600 and other *Klebsiella pneumoniae* strains through the formation of a fusion plasmid with another conjugative IncFIA plasmid. Fusion of these two plasmids could be mediated through interaction between the homologous region located in each plasmid, followed by homologous recombination (Xie et al., 2020).

In the present study we identified AP8555, a K1 serotype ST23 hypervirulent *K. pneumoniae* strain, collected from the blood sample of an ICU patient. The patient ultimately died due to the severity of infection caused by AP8555. This strain showed a hyperviscous phenotype in the string test. AP8555 exhibited typical features of hvKP and was demonstrated to be as virulent as the ST23 K1 serotype strain NUTH-K2044 in the killing assays conducted on mice and *G. mellonella.* The genome analysis showed that the AP8555 chromosome shared the same genetic background as the hvKP NTUH-K2044 strain. The sequence of the virulence plasmid pAP855 also harboured the same backbone as the multi-replicon IncHI1B/IncFIB plasmid in the hvKP NTUH-K2044 strain, which harbours major virulence genes such as *rmpA/rmpA2* and those encoding siderophores. Notably, compared with pK2044, pAP855 carried an additional and unique 130-Kb region associated with a conjugative IncFII(K) plasmid and harbouring multiple conjugation-related genes of T4SS and plasmid-mediated *CcdAB* TAS. The virulence plasmid pAP855 carry the 28-bp fusion site and the origin of the transfer site. Type 4 secretion system (T4SS) encoded by plasmids are involved in bacterial conjugation. We supposed this novel conjugative virulence plasmid perhaps formed of pK2044 and a conjugative InFII(K) plasmid similar to pN1863-FIIK due to replicative transposition. The *CcdAB* TAS has been extensively studied and is known to be involved in plasmid maintenance.

A comparison of this transfer region with those of other IncFII plasmids, revealed that several *tra* genes (*traJ*, *traY*, *traP*, and *traR*) as well as the *trbD, trbG, trbC, trbE, trbA*, and *trbJ* genes were missing (Nuk et al., 2011). Despite the incompleteness of the F-type T4SS, pAP855 was efficiently transferred *via* conjugation among *K. pneumoniae* strains in this study. However, our data suggest that the pAP855 imposed an extra burden on both ATCC700603 and J53, which partly hindered the prevalence of the plasmid. Within the limitation of the relatively small sample size, the results of the study highlight the need for future research to use more representative sample and provide important insight into the evolution of virulence-encoding elements during transmission in *K. pneumoniae* strains. The data obtained in this study reveal the pathogenic potential of this pathogen and highlight the need for surveillance and monitoring.

## Conclusion

Overall, we identified a cointegrate virulence plasmid pAP855 with high plasticity in an ST23 hvKP strain, belonging to the IncHI1B/IncFIB/IncFII incompatibility grouping. The plasmid could flexibly discard various regions and acquire the T4SS and TAS insertions, demonstrating its strong dissemination and evolution potential. Transferring this plasmid to clinic-associated strains could increase the virulence. Moreover, pAP855 is unstable in transconjugants under antibiotic-free conditions, indicating that the plasmid could not be stably inherited, possibly due to the large size of the plasmid. Virulence plasmids have certain stability and may cause nosocomial transmission in hospitals.

## Data availability statement

The datasets presented in this study can be found in online repositories. The names of the repository/repositories and accession number(s) can be found below: https://www.ncbi.nlm.nih.gov/nuccore/CP035383.1/.

## Ethics statement

The studies involving human participants were reviewed and approved by the Ethics Committee of the First Affiliated Hospital of Nanchang University. Patients involved in the study were anonymized, no informed consent was acquired because of the retrospective study. Written informed consent to participate in this study was provided by the participants’ legal guardian/next of kin. The animal study was reviewed and approved by the Ethics Committee of the First Affiliated Hospital of Nanchang University. Written informed consent was obtained from the owners for the participation of their animals in this study. Written informed consent was obtained from the individual(s), and minor(s)’ legal guardian/next of kin, for the publication of any potentially identifiable images or data included in this article.

## Author contributions

L-GW and T-XX did strain characterization and participated in manuscript writing. WZ, L-PF and YY conceived the study and performed data analyses. W-JL and T-XX did the whole-genome sequencing and comparative genomics and participated in manuscript writing. L-PF did the *G. mellonella* infection experiments. YL and D-DW wrote the paper. Q-SH collected the clinical data and analyzed the data. All authors contributed to the article and approved the submitted version.

## Funding

This study was supported by the National Natural Science Foundation of China (81860368, 82102411), Education Department of Jiangxi Province, China (GJJ160029), Jiangxi Science and Technology Department in China (20181BAB205065, 20202ACBL206025, 20202ACBL206023, and 20202ZDB01016), and Health and Family Planning Commission of Jiangxi Province (20188006 and 2018A330).

## Conflict of interest

The authors declare that the research was conducted in the absence of any commercial or financial relationships that could be construed as a potential conflict of interest.

## Publisher’s note

All claims expressed in this article are solely those of the authors and do not necessarily represent those of their affiliated organizations, or those of the publisher, the editors and the reviewers. Any product that may be evaluated in this article, or claim that may be made by its manufacturer, is not guaranteed or endorsed by the publisher.
