# Supplementary material for: Genetic characterization and passage instability of a novel hybrid virulence plasmid in a ST23 hypervirulent Klebsiella pneumoniae
Source: Front Cell Infect Microbiol. 2022 Jul 28;12:870779. doi: 10.3389/fcimb.2022.870779 (PMC9366888; doi:10.3389/fcimb.2022.870779)
Supplement: Supplementary file 1 [file DataSheet_1.doc]

**Genetic Characterization and Passage Instability of a novel Hybrid virulence Plasmid in a ST23 hypervirulent *Klebsiella pneumoniae***

**Lin-Ping Fan ^1，2^, Xiao-Yu Kong ^1，2^, Wenjian LIAO ^3^, Qi-Sen HUANG ^1，2^,Fang-Ling DU ^1^, Tian-xin XIANG^4^, Dan Dan WEI ^1^, La-Gen WAN^1^, Wei ZHANG^3^, and Yang LIU^1*^**

^1^Department of Clinical Microbiology, First Affiliated Hospital of Nanchang University, Nanchang University, Yong wai zheng jie No. 17, Nanchang, 330006, PR China

^2^School of Public Health, Nanchang University, Nanchang, Bayi Avenue No. 461, 330006, PR China

^3^Department of Respiratory and Critical Care Medicine, First Affiliated Hospital of Nanchang University, Nanchang University, Yong wai zheng jie No. 17, Nanchang, 330006, PR China

^4^Department of Infectious Disease, First Affiliated Hospital of Nanchang University, Nanchang University, Yong wai zheng jie No. 17, Nanchang, 330006, PR China

***Corresponding author:**

Yang Liu

First Affiliated Hospital of Nanchang University Yong wai zheng jie No. 17, Nanchang, 330006, PR China . Tel: 086-13576091584; Fax: 087-0791-88692794; e-mail: [ly13767160474@sina.com](mailto:ly13767160474@sina.com)

Running title: **A conjugative virulence plasmid in the K1 ST23 hvKP strain**

**Supplementary data**

**Supplementary Materials andMethods**

Ethics and consent

String test for the hypermucoviscosity phenotype

DNA preparation, genome sequencing, and annotation

**Table S1.** Minimal inhibitory concentrations (MICs)* for different antimicrobial agents of *K. pneumoniae* AP8555*, K. pneumoniae* NUTH-K2044*,* and *K. pneumoniae* ATCC700603

**Table S2** overall genomic features of AP8555

**Figure S1.** Positive hypermucoviscosity phenotype of *K. pneumoniae* AP8555 by string test.

**Figure S2**. (A) Schematic circular genome of *K. pneumoniae* AP8555 strain

1. genome comparison with other virulence plasmids

**Figure S3.** CPS production. CPS biosynthesis in the *K. pneumoniae* strains was determined by phenol-sulfuric acid assays.

**Supplementary Materials and Methods**

**Ethics and consent**

The present study is observational, and uses human wound sample for the ex *vivo* experiments. Sampling from the patient is routinely performed in the clinical treatment. The study protocol, including the procedure for obtaining verbal informed consent, was approved by the ethics committee of the first affiliated Hospital of Nanchang University, Jiangxi, China. Protocols of mouse experiments were approved by the same ethic committee.

**String test for the hypermucoviscosity phenotype**

The string test to determine the hypermucoviscosity phenotype was performed on colonies grown overnight on a blood agar plate at 37°C. The colonies were touched with a loop and pulled upwards, and a string of 5 mm or longer was considered to be a positive result.

**DNA preparation, genome sequencing, and annotation**

The genomic DNA of the strain AP8555 was extracted using the QIAamp DNA Mini Kit (Qiagen, Germany). The sequencing and assembly of the strain were carried out by Shanghai Yuanxu Biotechnology Co., ltd and described briefly below. A total of 5 µg genomic DNA from was sheared by g-TUBE (Covaris, US). The sequencing library with 10-kb size was constructed using the standard PacBio RS sample preparation instructions and then sequenced on Pacific Biosciences RS II sequencing platforms (Pacific Biosciences, US). Additionally, a 300-bp paired-end library from the same genomic DNA was prepared according to Illumina TruSeq DNA sample preparation recommendations and sequenced on Hiseq 2500 platforms (Illumina, US) with a read length of 150 bp. The Pacbio data (10 kb fragment library, 356,001 reads) were assembled using Hierarchical Genome Assembly Process (HGAP) software(Chin et al., 2013), generating a one-contig genome and four plasmids. The assembled genome and plasmids from the Pacbio data were further proofread using Hiseq data via Bowtie2 and samtools(Li et al., 2009; Langmead and Salzberg, 2012). Finally, a whole genome assembly without redundancy was obtained. Gene prediction for strain AP8555 was conducted using glimmer 3.02.(Delcher et al., 2007) tRNA and rRNA genes were predicted by tRNAscan-SE and RNAmmer, respectively(Schattner et al., 2005; Lagesen et al., 2007). The local blast was performed in a non-redundancy database download from NCBI (https://www.ncbi.nlm.nih.gov) to annotate the predicted genes. Sequence comparison was performed using the using BLAST analysis (http://blast.ncbi.nlm.nih.gov/Blast.cgi) and the BLAST Ring Image Generator v.0.95.22. Pathways involved in the genes were constructed by the use of Kyoto Encyclopedia of Genes and Genomes (KEGG)(Kanehisa and Goto, 2000). Gene COG was classified according to the conserved domain database(Marchler-Bauer et al., 2015). The STs and Capsular typing were determined by the website(https://bigsdb.pasteur.fr/klebsiella/klebsiella.html).The acquired antimicrobial resistance genes were identified by uploading assembled genomes to the Resfinder server v2.11. Putative virulence factors were predicted by VRprofile with the BLASTp-based *Ha*-value > 0.64, which collected 2,454 virulence factors from the Virulence Factors Database (VFDB). The genome was analyzed for the presence of prophages using PHAST(Zhou et al., 2011). The type VI secretion systems were also predicted by the VRprofile. CRISPR loci in the genome were identified by CRISPRFinder with the default arguments(Grissa et al., 2007). The plasmid replicons databases were obtained from the Center for Genomic Epidemiology (http://www. genomicepidemiology.org/).

**Table S1.** Minimal inhibitory concentrations (MICs) for different antimicrobial agents of *K. pneumoniae* NUTH-K2044*, K. pneumoniae* AP8555, *K. pneumoniae* ATCC700603, and *K. pneumoniae* ATCC700603-TC1

| Agent | NUTH-K2044 | | AP8555 | | ATCC700603 | | ATCC700603-TC1 | |
| --- | --- | --- | --- | --- | --- | --- | --- | --- |
|  | MIC | Interpretation | MIC | Interpretation | MIC | Interpretation | MIC | Interpretation |
| Ampicillin | >256 | R | >256 | R | >256 | R | >256 | R |
| Piperacillin | ≤16 | S | ≤16 | S | >256 | R | >256 | R |
| Ampicillin/ sulbactam | ≤8/4 | S | ≤8/4 | S | 32/16 | R | 32/16 | R |
| Piperacillin/ tazobactam | ≤16/4 | S | ≤16/4 | S | 32/4 | I | 32/4 | I |
| Cefazolin | ≤16 | S | ≤16 | S | 64 | R | 64 | R |
| Cefuroxime | ≤8 | S | ≤8 | S | 128 | R | 128 | R |
| Cefoxitin | ≤8 | S | ≤8 | S | 128 | R | 128 | R |
| Cefotaxime | ≤1 | S | ≤1 | S | 64 | R | 64 | R |
| Ceftazidime | ≤4 | S | ≤4 | S | 32 | R | 32 | R |
| Ceftriaxone | ≤1 | S | ≤1 | S | 64 | R | 64 | R |
| Cefepime | ≤ 2 | S | ≤ 2 | S | 16 | R | 16 | R |
| Aztreonam | ≤4 | S | ≤4 | S | 32 | R | 32 | R |
| Ertapenem | ≤0.5 | S | ≤0.5 | S | ≤0.5 | S | ≤0.5 | S |
| Imipenem | ≤1 | S | ≤1 | S | ≤1 | S | ≤1 | S |
| Meropenem | ≤1 | S | ≤1 | S | ≤1 | S | ≤1 | S |
| Gentamicin | ≤4 | S | ≤4 | S | ≤ 2 | S | ≤ 2 | S |
| Amikacin | ≤16 | S | ≤16 | S | ≤ 1 | S | ≤ 1 | S |
| Ciprofloxacin | ≤1 | S | ≤1 | S | ≤0.25 | S | ≤0.25 | S |
| Levofloxacin | ≤1 | S | ≤1 | S | ≤ 1 | S | ≤ 1 | S |
| Trimethoprim/ sulfamethoxazole | ≤2/38 | S | ≤2/38 | S | ≤2/38 | S | ≤2/38 | S |
| Tetracycline | ≤4 | S | ≤4 | S | ≤4 | S | ≤4 | S |
| Tigecycline | ≤1 | S | ≤1 | S | ≤1 | S | ≤1 | S |

**Table S2 overall genomic features of AP8555**

| Parameter | Chromosome of AP8555 | pAP855 |
| --- | --- | --- |
| Size(bp) | 5463931 | 357837 |
| G+C(%) | 57.45 | 51.01 |
| No. Of predicted ORFs | 5123 | 351 |
| Resistance gene(s) | fosA blaSHV-190 oqxA, oqxB | None |
| Plasmid replicon | None | IncHI1B  IncFII(k)  IncFIBK |


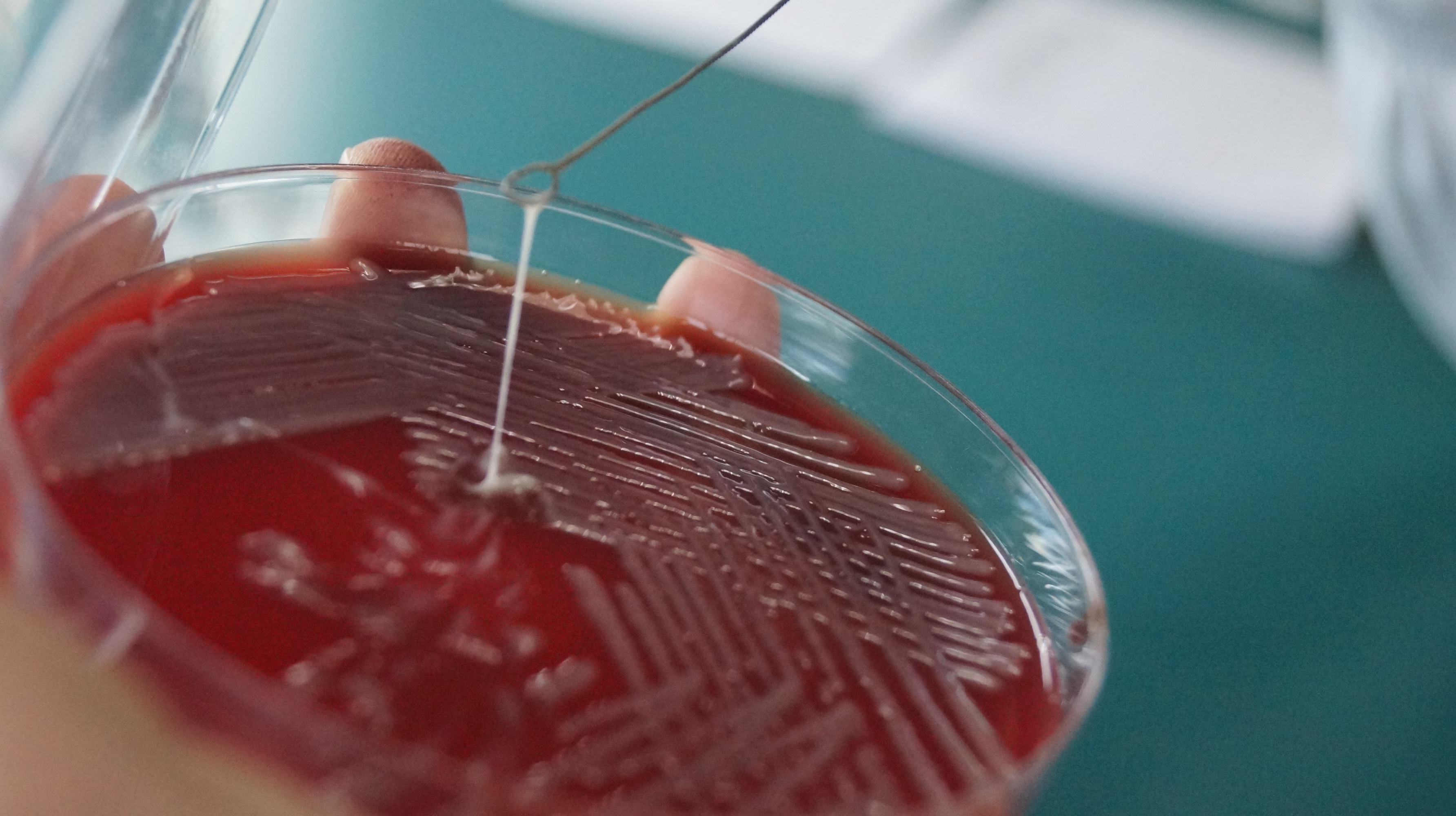

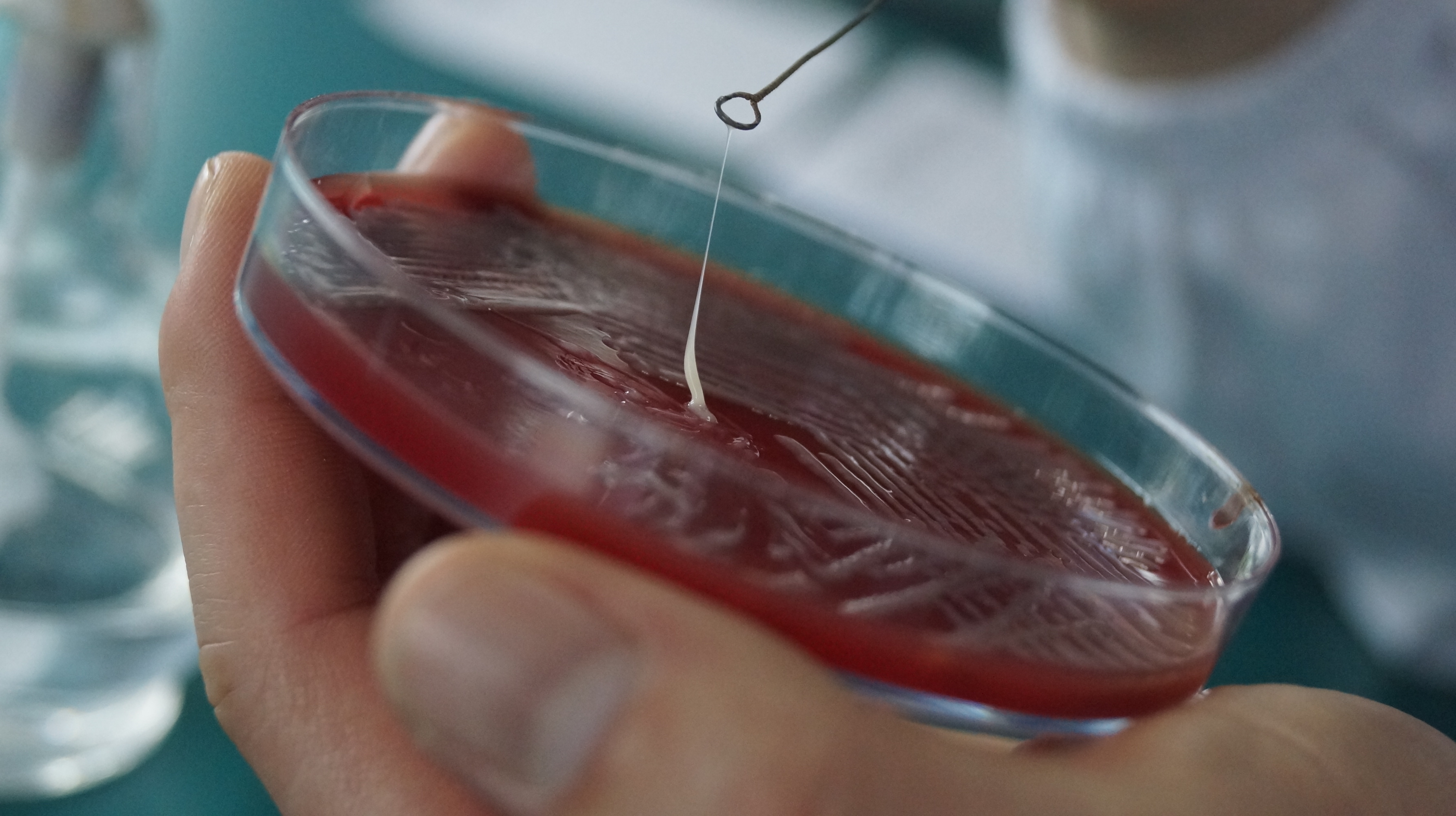

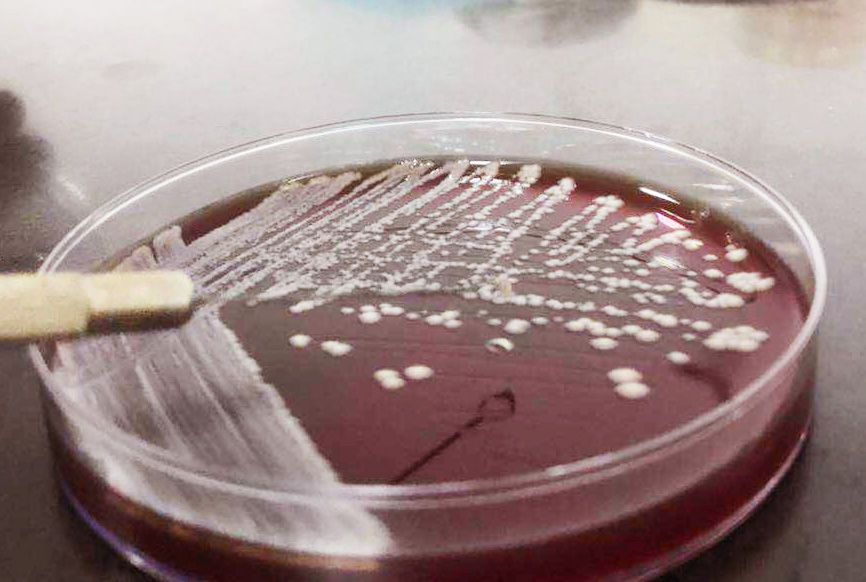


**AP8555**

**NTUH-K2044**

**ATCC700603**


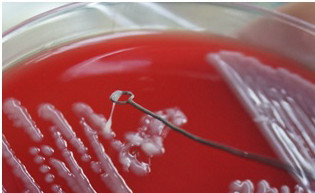


**ATCC700603-TC1**

**Figure S1.** **Positive hypermucoviscosity phenotype of *K. pneumoniae* AP8555 by string test.**


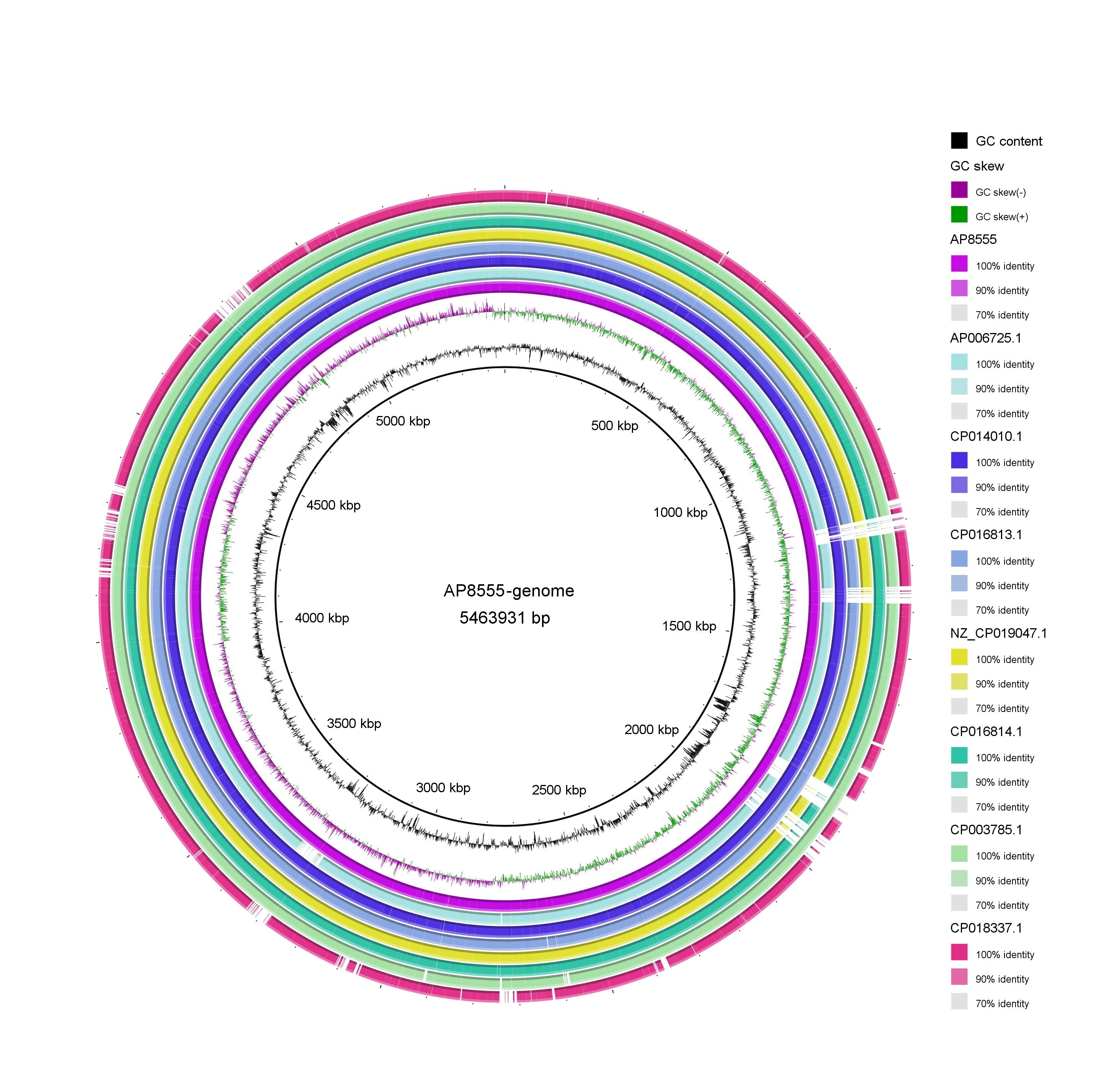


**
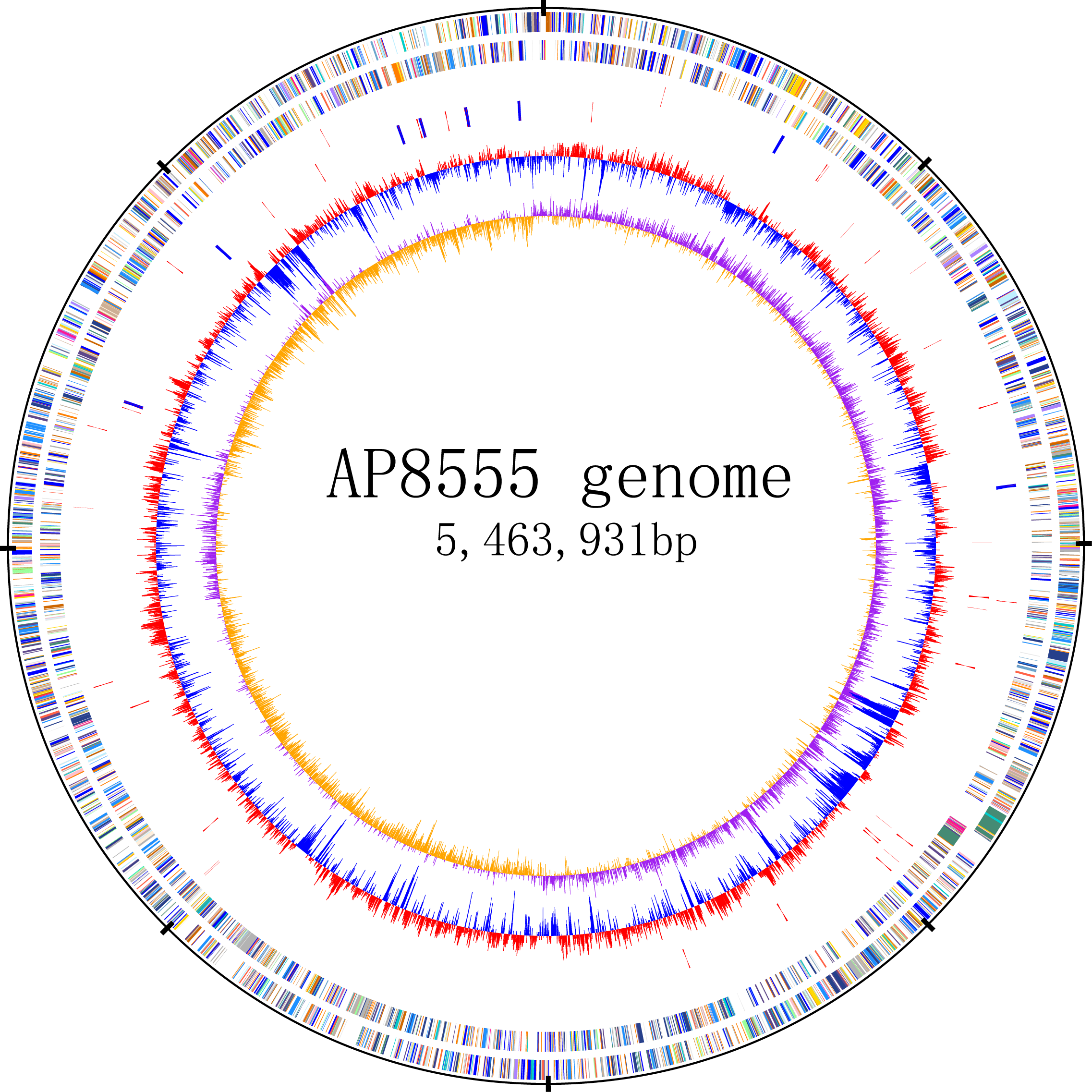
**

.


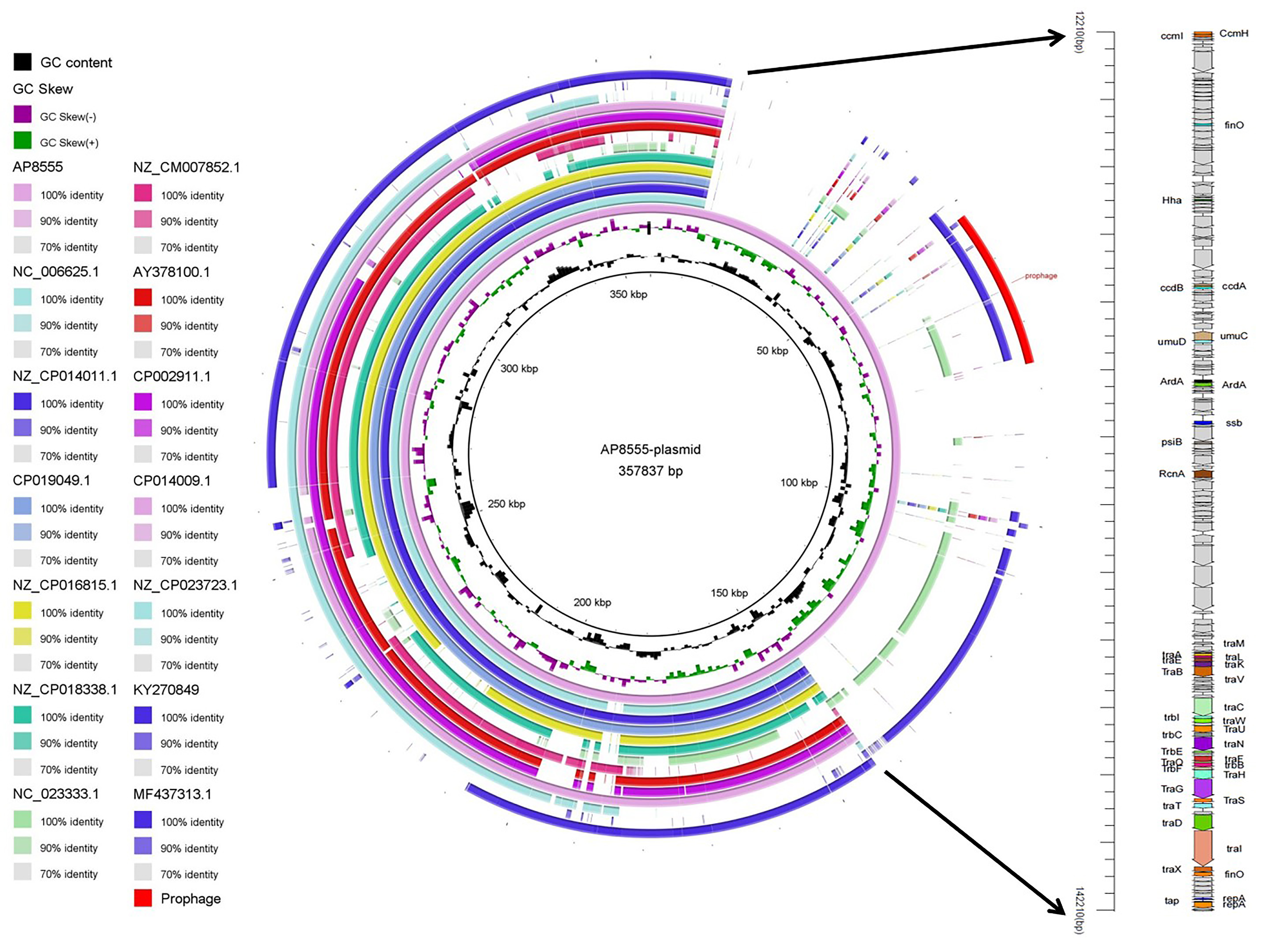


**Figure S2**. **A Schematic circular genome and genome comparison with other hvKP strains**

**B genome comparison with other virulence plasmids**

**
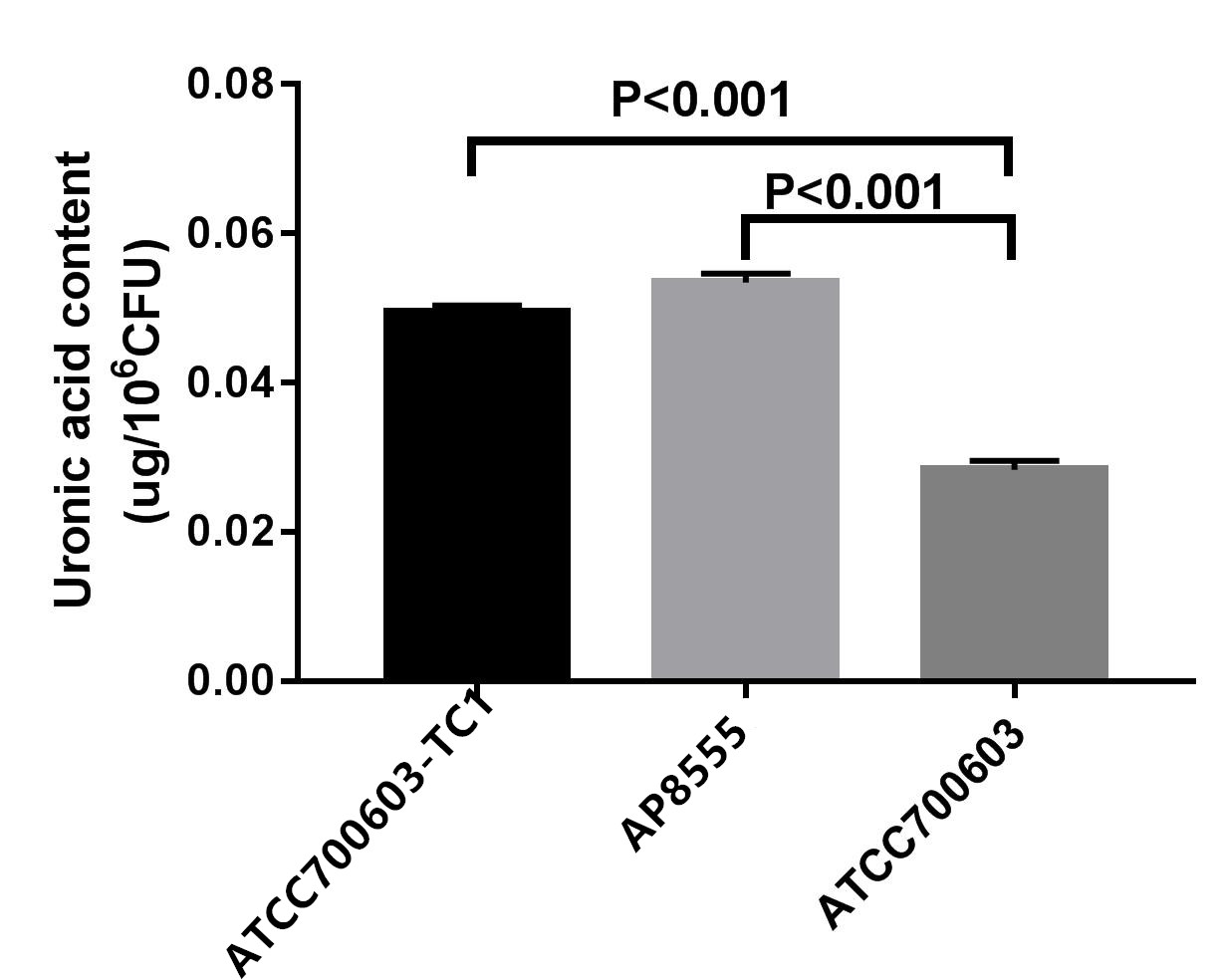
**

**Figure S3** **CPS production. CPS biosynthesis in the *K. pneumoniae* strains was determined by phenol-sulfuric acid assays.**

*, *P* < 0.05 versus the value for a corresponding cKP reference strain ATCC700603.

Error bars indicate the standard deviations for three triplicate samples.

REFERENCES

Chin, C. S., Alexander, D. H., Marks, P., Klammer, A. A., Drake, J., Heiner, C., et al. (2013). Nonhybrid, finished microbial genome assemblies from long-read SMRT sequencing data. *Nat. Methods* 10, 563-569. doi: 10.1038/nmeth.2474

Delcher, A. L., Bratke, K. A., Powers, E. C., and Salzberg, S. L. (2007). Identifying bacterial genes and endosymbiont DNA with Glimmer. *Bioinformatics* 23, 673-679. doi: 10.1093/bioinformatics/btm009

Grissa, I., Vergnaud, G., and Pourcel, C. (2007). CRISPRFinder: a web tool to identify clustered regularly interspaced short palindromic repeats. *Nucleic Acids Res.* 35, W52-57. doi: 10.1093/nar/gkm360

Kanehisa, M., and Goto, S. (2000). KEGG: kyoto encyclopedia of genes and genomes. *Nucleic Acids Res.* 28, 27-30. doi: 10.1093/nar/28.1.27

Lagesen, K., Hallin, P., Rødland, E. A., Staerfeldt, H. H., Rognes, T., and Ussery, D. W. (2007). RNAmmer: consistent and rapid annotation of ribosomal RNA genes. *Nucleic Acids Res.* 35, 3100-3108. doi: 10.1093/nar/gkm160

Langmead, B., and Salzberg, S. L. (2012). Fast gapped-read alignment with Bowtie 2. *Nat. Methods* 9, 357-359. doi: 10.1038/nmeth.1923

Li, H., Handsaker, B., Wysoker, A., Fennell, T., Ruan, J., Homer, N., et al. (2009). The Sequence Alignment/Map format and SAMtools. *Bioinformatics* 25, 2078-2079. doi: 10.1093/bioinformatics/btp352

Marchler-Bauer, A., Derbyshire, M. K., Gonzales, N. R., Lu, S., Chitsaz, F., Geer, L. Y., et al. (2015). CDD: NCBI's conserved domain database. *Nucleic Acids Res.* 43, D222-226. doi: 10.1093/nar/gku1221

Schattner, P., Brooks, A. N., and Lowe, T. M. (2005). The tRNAscan-SE, snoscan and snoGPS web servers for the detection of tRNAs and snoRNAs. *Nucleic Acids Res.* 33, W686-689. doi: 10.1093/nar/gki366

Zhou, Y., Liang, Y., Lynch, K. H., Dennis, J. J., and Wishart, D. S. (2011). PHAST: a fast phage search tool. *Nucleic Acids Res.* 39, W347-352. doi: 10.1093/nar/gkr485
